# Identifying associations and key early life exposure windows for organophosphate esters and adolescent brain morphometry in the HOME study

**DOI:** 10.1016/j.envres.2026.124509

**Published:** 2026-04-13

**Authors:** Zheng Ren, Aimin Chen, Jagadeesh Puvvula, Yingying Xu, Ethan Bouche, Antonia M. Calafat, Alex D. Edmondson, Kristin A. Linn, Russell T. Shinohara, Maria Ospina, Zana Percy, Ann M. Vuong, Joseph M. Braun, Bruce P. Lanphear, Kimberly Yolton, Quy Cao, Kim M. Cecil

**Affiliations:** a Department of Biostatistics, Johns Hopkins Bloomberg School of Public Health, USA; b Penn Statistics in Imaging and Visualization Center, Department of Biostatistics, Epidemiology, and Informatics, University of Pennsylvania, Philadelphia, PA, USA; c Department of Biostatistics, Epidemiology and Informatics, Perelman School of Medicine, University of Pennsylvania, Philadelphia, PA, USA; d Department of Pediatrics, Cincinnati Children’s Hospital Medical Center, University of Cincinnati College of Medicine, Cincinnati, OH, USA; e National Center for Environmental Health, U.S. Centers for Disease Control and Prevention, Atlanta, GA, USA; f Department of Radiology, Cincinnati Children’s Hospital Medical Center, University of Cincinnati College of Medicine, Cincinnati, OH, USA; g Department of Environmental & Public Health Sciences, College of Medicine, University of Cincinnati, Cincinnati, OH, USA; h Center for Biomedical Image Computing and Analytics, University of Pennsylvania, Philadelphia, PA, USA; i Department of Epidemiology and Biostatistics, School of Public Health, University of Nevada, Las Vegas, Las Vegas, NV, USA; j Department of Epidemiology, Brown University, Providence, RI, USA; k Faculty of Health Sciences, Simon Fraser University, Burnaby, BC, Canada; l Child and Family Research Institute, BC Children’s Hospital, Vancouver, BC, Canada

**Keywords:** Organophosphate esters, Brain structure, Neurochemistry, Child, Neurobehavior, Grooved pegboard

## Abstract

**Background:**

Organophosphate esters (OPEs) can be used as flame retardants and plasticizers in consumer products. Early life exposure may disrupt brain development by modifying brain structure and neurochemistry.

**Objectives:**

To evaluate the associations between gestational and childhood OPE metabolite urinary concentrations and their mixtures with neurobehavioral outcomes, explore the potential mediation of brain structure and neurochemistry, and identify potential windows of susceptibility.

**Methods:**

We employed quantile-based g-computation models to investigate the mixture associations of OPE metabolite concentrations with three distinct categories of outcomes within the HOME Study at age 12 years: 1) magnetic resonance imaging (MRI)-based morphometric measurements; 2) magnetic resonance spectroscopy (MRS)-derived neurochemical concentrations; and 3) neurobehavioral outcomes across four non-overlapping exposure windows: 1) gestational, 2) birth, 3) ages 1–3 years, and 4) ages 5–8 years. We identified outcomes associated with OPE metabolite concentrations. We conducted analyses to evaluate the potential mediation of changes in brain structure and neurochemistry.

**Results:**

Urinary concentrations of the mixture of OPE metabolites were primarily associated with morphometric measurements compared to neurobehavioral outcomes and were seen across all windows investigated before adjusting for multiple comparisons. The OPE metabolite mixture in the 5–8 year window was associated with reduced cortical volume of the pars triangularis, which mediated 27% of the increase in dominant hand drops on the grooved pegboard test before adjusting for multiple comparisons.

**Discussion:**

Cortical volume of the pars triangularis may mediate the adverse relationship between OPE metabolite mixture concentrations and adolescent visual motor function during the 5–8-year window.

## 1. Introduction

Flame retardants are primarily used in the production of consumer products (plastics, textiles, and furnishing foam) to minimize fire hazards associated with the combustion of polymeric material (Miller and Martin, 1978). Since the mid-2000s, the usage of organophosphate esters (OPEs) as flame retardants has increased (Blum et al., 2019; Dodson et al., 2012). Some OPEs are applied to materials without chemical bonding; thus, they typically leach into the environment (van der Veen and de Boer, 2012).

Exposures to OPEs occur primarily through the ingestion of indoor dust (Peng et al., 2023). A representative sample of the general population of the USA age 6 years and older detected OPE metabolites, diphenyl phosphate (DPHP), bis(1,3-dichloro-2-propyl) phosphate (BDCIPP), bis-(1-chloro-2-propyl) phosphate (BCIPP), bis-2-chloroethyl phosphate (BCEP), di-n-butyl phosphate (DNBP), in up to 80% of study participants (Ospina et al., 2018). Exposures to OPEs at environmentally relevant doses may alter neurobehavior by interfering with noncholinergic pathways, disrupting neurotransmitter concentrations such as glutamate (GLU) and gamma-aminobutyric acid (GABA) (He et al., 2024; Patisaul et al., 2021). Impaired mitochondrial processes, including oxidative phosphorylation, respiratory electron transport chain assembly and function, were observed in an animal model with developmental exposure to OPEs (Witchey et al., 2023). Oxidative stress can damage mitochondrial and nuclear DNA along with lipid peroxidation (Ingle et al., 2020). OPE metabolites in pregnant women were associated with increases in biomarkers of oxidative stress (Ingle et al., 2020). Additionally, several population-based studies have suggested adverse associations between OPE metabolites and neurobehavioral outcomes (Castorina et al., 2017; Doherty et al. 2019a, 2019b; Hernandez-Castro et al., 2023; Lipscomb et al., 2017; Liu et al., 2021).

It is unknown, however, whether OPE exposure during early brain development is associated with changes in early adolescent brain structure or intrinsic neurochemistry, and if such changes would mediate the relationship between OPEs and neurobehavior (Cecil, 2022). To address this gap, we evaluated the associations between mixtures of gestational and childhood OPE metabolite urinary concentrations and neurobehavioral outcomes, explored the potential mediation of magnetic resonance imaging (MRI)-derived morphometric measurements (e.g., volume, cortical thickness) of the brain and magnetic resonance spectroscopy (MRS)-derived intrinsic neurochemical concentrations, and identified potential windows of susceptibility in early life. Neuroimaging and neurobehavioral assessments targeted at age 12 years allowed us to assess a critical neurodevelopmental time-point, the beginning of adolescence, as synaptic pruning, myelination, and neuronal reorganization adapts to the significant neurobiological processes associated with puberty.

## 2. Methods

### 2.1. Study participants

We analyzed data from the Health Outcomes and Measures of the Environment (HOME) Study, an ongoing prospective pregnancy and birth cohort established in the Cincinnati metropolitan area (Ohio, USA), recruited between 2003 and 2006, to investigate how prevalent environmental exposures influence child health and development. Detailed information on enrollment, inclusion, and exclusion criteria, as well as assessments of environmental chemical exposures and neurobehavioral assessments, has been described previously (Braun et al. 2017, 2020). A total of 420 children (11 sets of twins included) completed at least one subsequent study visit from birth to age 12 years. For the 12-year study visit, 256 caregiver-adolescent pairs completed the visit. All data collection was completed prior to the COVID-19 pandemic.

The institutional review boards at Cincinnati Children’s Hospital Medical Center (CCHMC) and the enrolling delivery hospitals approved this study. The Centers for Disease Control and Prevention (CDC) laboratory’s involvement did not constitute engagement in human-subjects research. Caregivers provided written informed consent for their own participation, as well as for their child’s. At age 12 years, adolescents provided written informed assent.

### 2.2. OPE metabolites measurements

We collected maternal urine samples during pregnancy (at 16 weeks, 26 weeks, and the birth visit) and child urine samples up to 5 times (at ages 1, 2, 3, 5, and 8 years). Urine samples from pregnant women and toilet-trained children were collected in polypropylene specimen cups, whereas urine samples from non-toilet-trained children were collected using Kendall’s abdominal pads (Yang et al., 2024). These urine samples were analyzed for four OPE metabolites: 1) BDCIPP; 2) BCEP; 3) DPHP; and 4) DNBP using isotope dilution high-performance liquid chromatography-tandem mass spectrometry; the limit of detection (LOD) was 0.1 μg/L for the four OPE metabolites; the majority of the metabolites were detected in at least 80% of samples (Supplementary Table S1) (Jayatilaka et al., 2019). Additionally, urine-specific gravity was quantified using a hand-held refractometer (Atago model PAL-10 S, Tokyo, Japan (Yang et al., 2024)).

We first imputed metabolite concentrations below LOD with LOD divided by the square root of 2 for descriptive statistics (Hornung and Reed, 1990). We then replaced the values below the LOD with a random value within the LOD range (0 - LOD) using the fill-in single imputation method, following a left-truncated distribution with the derived distribution parameters (Lubin et al., 2004). Upon imputing the OPE metabolite concentrations below the LOD, we standardized the metabolite concentrations using urine specific gravity (Hauser et al., 2004):

$$\text{STD}\_\text{OP}E_{\text{urine}}=\text{concentratio}n_{i}\frac{\left(SG_{m}–1\right)}{\left(SG_{i}–1\right)}$$

where *STD_OPE*_*urine*_ is the specific-gravity standardized urinary OPE metabolite concentrations, *concentration*_*i*_ is the urinary concentration of each OPE biomarker measured for the i^th^ observation, *SG*_*m*_ is the median urinary specific gravity of the participant pool at a given time point, and *SG*_*i*_ is the urinary specific gravity of the i^th^ participant.

### 2.3. Primary outcome categories

We measured three distinct categories of outcomes assessed at age 12 years: 1) MRI-based morphometric measurements, 2) MRS-based neurochemical concentrations, and 3) neurobehavioral outcomes assessing memory, motor, executive functioning, and internalizing and externalizing behaviors.

#### 2.3.1. MRI and MRS acquisition

MRI and MRS data were acquired using a Philips Ingenia MR scanner operating at 3 T field strength and equipped with a 32-channel head coil. A three-dimensional (3D), high-resolution, isotropic, T1-weighted fast Fourier echo anatomical imaging sequence was performed using 8.2 ms (ms) repetition time, 3.7 ms echo time, 1057 ms inversion time, 8-degree flip angle, sensitivity encoding factor of 2, contiguous slices with a 1 mm thickness, and 1 mm × 1 mm voxel size. For MRS acquisitions, a single voxel, point resolved spectroscopy (PRESS) sequence was employed using a 2000 ms repetition time, 30 ms echo time, and 128 averages with an excitation water suppression method (window of 140 Hz, 300 s pulse angle) along with an embedded unsuppressed water reference series of 16 averages. The eight cubic centimeter voxel for MRS was prescribed about the perigenual anterior cingulate cortex within the medial frontal lobe, localized from the 3D T1-weighted imaging sequence.

#### 2.3.2. MRI morphometric data processing

FreeSurfer software (version 6.0.0, Laboratory for Computational Neuroimaging, Athinoula A. Martinos Center for Biomedical Imaging, Charlestown, Massachusetts; http://surfer.nmr.mgh.harvard.edu/) was used to process anatomical T1-weighted images into morphometric measurements employing the recon-all function. FreeSurfer is an optimized method for cortical reconstruction and volumetric segmentation (Dale et al., 1999; Dale and Sereno, 1993; Desikan et al., 2006; Fischl and Dale, 2000; Fischl et al. 1999a, 1999b, 2001, 2002, 2004a, 2004b; Han et al., 2006; Jovicich et al., 2006; Reuter et al., 2010; Segonne et al. 2004, 2007; Sled et al., 1998). Upon completion, FreeSurfer yielded robust measures of cortical volume, subcortical volume, and cortical thickness for each participant based on the Desikan-Killiany atlas (Desikan et al., 2006). The initial FreeSurfer output included 60 subcortical volumes, and for each hemisphere, 34 cortical volumes and 34 cortical thickness measures. In cases where specific morphometric measurements existed in both the left and right hemispheres and demonstrated a strong positive linear correlation exceeding 0.5, we computed their average value as a surrogate for the morphometric measurement; otherwise, we treated them as separate measurements by hemisphere. Following hemispheric aggregation and exclusion of subcortical regions with unreliable or redundant measurements, the final analytic dataset included 25 subcortical volumes, 38 cortical volumes, and 44 cortical thickness measures per participant.

#### 2.3.3. MRS data processing

The raw spectroscopy data were processed using LCModel software (Provencher, 1993) to determine the neurochemical signal areas relative to the unsuppressed water signal area. To convert neurochemical signals into millimolar (mM) concentration units, we accounted for the tissue composition of the voxel sampled and relaxation rates for water and individual neurochemicals, based on literature-determined values. Using custom code written in Python, we called upon the FMRIB Software Library (FSL, http://www.fmrib.ox.ac.uk/fsl) brain extraction and segmentation tools to segment each T1 image and calculate the percentage of each tissue type (grey matter, white matter, and cerebrospinal fluid) within each MRS voxel. The raw neurochemical concentrations were adjusted for the tissue contributions (Woolrich et al., 2009), and for the T1 and T2 relaxation decay rates of the tissue-corrected water concentration and primary neurochemicals, including N-acetyl aspartate (NAA), choline (Cho), creatine (Cr), and myo-inositol (mI) (Tal et al., 2012; Traber et al., 2004; Wansapura et al., 1999). However, GLU alone, and composite glutamine and glutamate (GLX) concentrations were corrected for tissue contribution and water relaxation but remained unadjusted for neurochemical T1 and T2 relaxation decay due to the inability to resolve glutamine from GLU signals at 3 T (Gussew et al., 2012). This produced six intrinsic neurochemical concentrations for each participant from the perigenual anterior cingulate cortex.

#### 2.3.4. Neurobehavioral assessments

Neurobehavioral assessments at age 12 years encompassed a range of domains, including outcomes from the Child and Adolescent Memory Profile (ChAMP) (Sherman et al., 2015), grooved pegboard test (GPT) (Lafayette Instrument, 2014), Behavior Rating Inventory of Executive Function, Second Edition (BRIEF-2) (Gioia et al., 2015), and the Behavior Assessment System for Children, Third Edition (BASC-3) (Reynolds and Kamphaus, 2015). The tests were administered to each participant by a research coordinator trained in proper administration and scoring. Assessments took place in a quiet room free from distractions in our study clinic (Piehl et al., 2016). We briefly summarize these assessments.

The ChAMP is a comprehensive, norm-referenced test of memory and learning that produces the following indexes: Verbal Memory, Visual Memory, Immediate Memory, Delayed Memory, and Total Memory (Sherman et al., 2015). All these scores were expressed as standard scores (M = 100, SD = 15), with higher scores reflecting better performance. The 10 ChAMP subtests are based on four tasks, two of which involved verbal stimuli and two that involved visual stimuli.

The GPT assesses manual dexterity using rapid visual motor coordination and psychomotor speed. Dexterity is the “manual ability that requires rapid coordination of gross and fine voluntary movement, based on a certain number of capacities that are developed through learning, training, and experience” (Poirier, 1988). The GPT involves the rapid, one-at-a-time insertion of 25 keyhole-shaped pegs (1-inch metal) into matching grooves (a board with a 5-by-5 array of grooves, with each groove at a different angle than adjacent grooves in the row) (Bryden and Roy, 2005). Participants were instructed to insert all pegs into the holes, one row at a time, without skipping any, using one hand at a time, starting with their dominant hand (DH) and followed by their nondominant hand (NDH) (Ruff and Parker, 1993). The time required to place all the pegs and the number of dropped pegs using each hand were included as outcomes.

We assessed adolescent executive function using the BRIEF-2 (Gioia et al., 2015), a well-validated assessment of executive function within the context of everyday functioning. We used both caregiver-report (63 items) and adolescent self-report (55 items) versions, which provide T-scores for a Global Executive Composite (GEC) and three indexes: Behavior Regulation Index (BRI), Emotion Regulation Index (ERI), and Cognitive Regulation Index (CRI), as well as subscales including inhibit, shift, emotional control, working memory, plan/organize, and monitor. Additional scales on the caregiver version include initiation, organization of materials, and task monitoring; task completion is an additional scale on the self-report version. T-scores have a mean of 50 and a standard deviation of 10, with higher scores indicating greater deficits in executive function. A BRIEF T-score of 65 or above is considered potentially clinically elevated (Baron, 2000).

We used the caregiver-report and self-report versions of the BASC-3, a valid and reliable measure of children’s adaptive and problem behaviors in community and home settings (Reynolds and Kamphaus, 2015). Composite scores were derived using the publisher’s online scoring program. Composite indexes for adolescent self-reports included assessments for Emotional Symptoms (ESI), Functional Impairment (FII), Inattention/Hyperactivity (IHI), Internalizing Problems (INZ), Personal Adjustment (PAI), and School Problems (SPI), each with clinical subscales. Composite indexes for caregiver reports included assessments for Adaptive Skills, Externalizing Problems, Internalizing Problems, and Behavior Symptoms, each with clinical subscales. We also selectively included the content scales for Executive Functioning and Resiliency. Scores were sex-combined and normalized to a mean (± standard deviation (SD)) of 50 ± 10.

### 2.4. Analytical design

In this study, we first investigated the associations between OPE metabolite concentrations and neurochemical concentrations, brain morphometry, and neurobehavioral outcomes across six overlapping subgroups with slight differences in sample size due to missing data: the Morphometry Subgroup, MRS Subgroup, ChAMP Subgroup, GPT Subgroup, BRIEF Subgroup, and BASC Subgroup. In a later stage, to investigate the mediation associations of OPE metabolites on neurobehavioral outcomes through changes in brain morphometry or neurochemical concentrations, we created four combined subgroups by selecting participants who were included in both the Morphometry Subgroup and MRS Subgroup, and one of the four types of Neurobehavior Subgroups. These four subgroups included: Mediation: ChAMP Subgroup, Mediation: GPT Subgroup, Mediation: BRIEF Subgroup, and Mediation: BASC Subgroup.

### 2.5. Confounding variables

We identified and selected five confounding variables to adjust for in all regression models based on prior analyses in the HOME Study evaluating OPE exposure and neurobehavioral outcomes (Percy et al. 2021, 2022, 2023). These included maternal race, maternal education, gestational exposure to cigarette smoke, as measured by maternal serum cotinine during pregnancy, child sex, and Home Observation for Measurement of the Environment score at age 12 months (Bradley and Caldwell, 1980). We categorized maternal race as non-Hispanic white or other, and maternal education as High School or below, some college, bachelor’s degree, or graduate school. Additionally, when examining morphometric measurements as the outcome, we included total intracranial volume (ICV) as a covariate to account for differences in head size among individuals.

### 2.6. Identification of windows of susceptibility

In this study, urinary concentrations of OPE metabolites were quantified at eight different time points that were then summarized into four non-overlapping windows: 1) gestational, 2) birth, 3) 1–3 years, and 4) 5–8 years. We estimated window-specific OPE exposures by calculating the average OPE metabolite concentration within each window, except at birth, when only one urine sample was collected from each mother.

### 2.7. Statistical methods

**Fig. 1.**
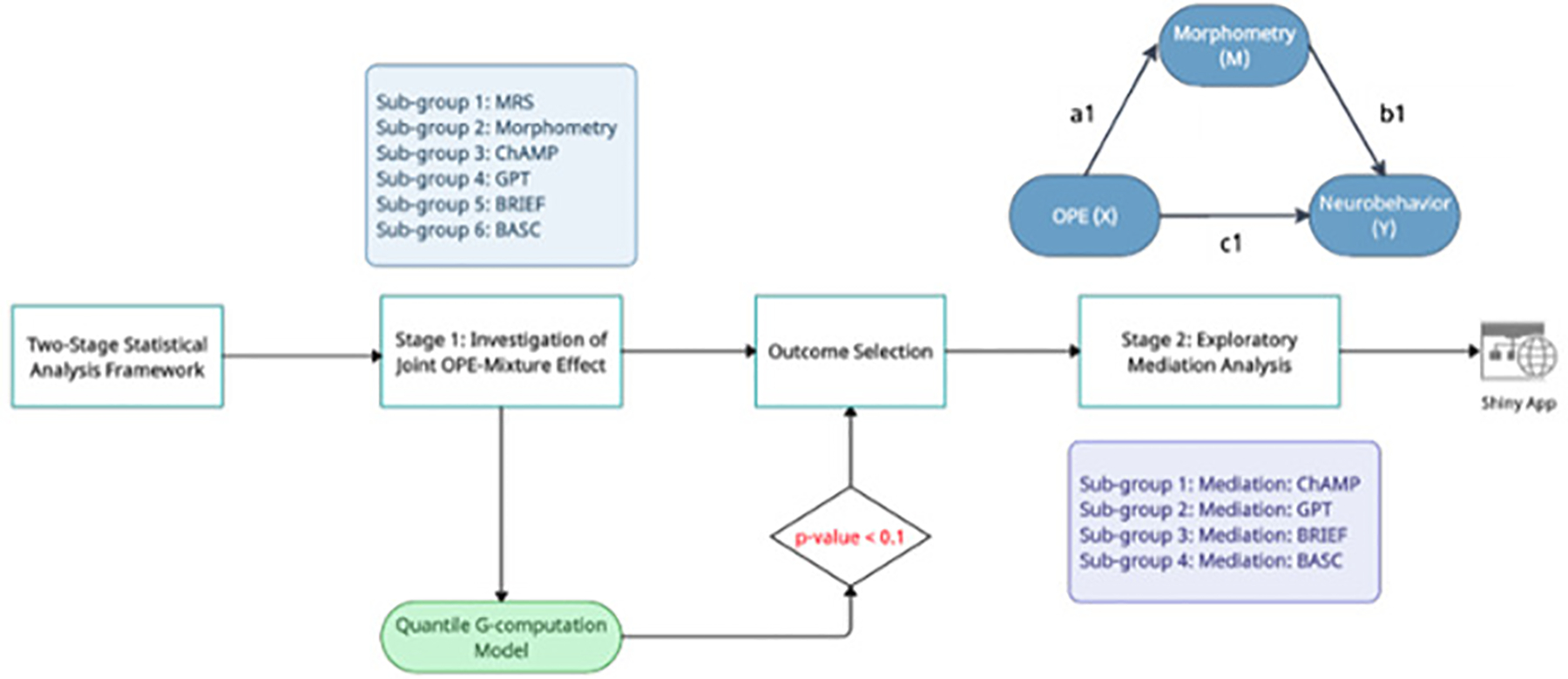
A two-stage statistical analysis framework for exploring the relationship between organophosphate esters (OPEs) and neurobehavior. Stage 1 involves a quantile g-computation model across six distinct subgroups to investigate joint associations of OPE metabolites with adolescents’ morphometric measurements, MRS-derived neurochemical concentrations, and neurobehavioral outcomes across different time windows. Stage 2 consists of an exploratory mediation analysis across four combined subgroups, assessing the direct (c_1_) and indirect associations (a_1_*b_1_) of OPE on neurobehavior, with morphometry as a potential mediator. The results are integrated into a Shiny app for interactive exploration.

We adopted a two-stage analytical approach (Fig. 1) to comprehensively explore the potential associations between OPE metabolite concentrations and adolescents’ morphometric measurements, MRS-derived neurochemical concentrations, and neurobehavioral outcomes across different exposure time windows. Specifically, we investigated both the direct associations between OPE metabolite concentrations and neurobehavioral outcomes, and explored potential indirect associations mediated by changes in morphometry or neurochemical concentrations.

#### 2.7.1. Stage 1: investigation of joint OPE mixture

In this stage, we explored the associations of all four OPE metabolites on the adolescent’s morphometric measurements, neurochemical concentrations, and neurobehavioral outcomes across four non-overlapping windows: gestational, birth, 1–3 year, and 5–8 year. To address severe outliers in outcome variables that significantly impact model fits, we employed the 1.5 IQR method to exclude them, constituting less than 5% of our data. The method identifies outliers as values outside *Q*1 – 1.5 × *IQR* or *Q*3+ 1.5 × *IQR*, where IQR represents the interquartile range, and Q1 and Q3 are the 25th and 75th percentiles, respectively.

We applied the quantile-based g-computation model to estimate the joint association of all four OPE metabolites as a mixture in the same window, using separate models for the four non-overlapping windows (Keil et al., 2020). This method recategorized the OPE metabolite concentrations into quantiles to estimate the overall association of simultaneously increasing all OPE metabolites with the outcome of interest. Accordingly, quantile g-computation was used to evaluate the joint association of the OPE metabolite mixture with the outcome of interest, while also allowing assessment of the relative contribution of each OPE metabolite. For individual i, the model can be expressed as

$$Y=\beta_{0}+\sum_{j=1}^{4}\beta_{j}X_{ji}^{\left(q\right)}+\gamma^{⊤}C_{i}+\varepsilon_{i}$$

Where *Y*_*i*_ denotes the outcome, *β*_0_ is the model intercept, $X_{ji}^{\left(q\right)}$ denotes the j^th^ OPE metabolite transformed into quantiles, *β*_*j*_ is the corresponding regression coefficient, *C*_*i*_ is the vector of covariates with coefficient vector γ, and *ε*_*i*_ is the error term. In our analysis, we divided the OPE metabolite concentrations into ten quantiles (q = 10). The overall joint association of the OPE mixture with the outcome was summarized by the sum of the component coefficients, $\varphi =\sum_{j=1}^{4}\beta_{j}$. In this study, we focused primarily on this overall joint effect, although the relative contribution of each metabolite can also be characterized using the estimated component weights.

Linear regression models were used for continuous outcomes, and Poisson regression models were used for positive integer outcomes, which include the number of drops during GPT tasks, using both the non-dominant and dominant hands. We employed the Benjamini-Hochberg (BH) procedure (Benjamini and Hochberg, 1995) to control the false discovery rate across all models fitted in the initial screening stage, this approach was selected to retain potentially meaningful signals given the large number of tests.

#### 2.7.2. Stage 2: exploratory mediation analysis

Following the first stage, we conducted mediation analyses for all combinations of mediators and neurobehavioral outcomes within each period among associations with unadjusted p-values <0.1 in the prior analysis. These findings were treated as exploratory. Specifically, mediation analyses investigate whether the association between joint OPE metabolite concentration (X) and neurobehavioral outcomes (Y) can be mediated by morphometric measurement or neurochemical concentrations (M) (Geldhof et al., 2017) (Fig. 1). All mediation models were adjusted for a set of relevant covariates (C) given the mediator (M). The mediation framework can be described as:

$$M=a_{0}+a_{1}X+\gamma^{⊤}C+\varepsilon_{M}$$

$$Y=b_{0}+b_{1}M+c_{1}X+\delta^{⊤}C+\varepsilon_{Y},$$

where Y denotes the outcome variable, X represents the OPE mixture index constructed from metabolites categorized into deciles using quantile g-computation, M denotes the mediator, and C represents the matrix of covariates included in both models. The indirect association is calculated based on the product of *a*_1_ × *b*_1_, and the total association from OPE mixture to neurobehavioral outcome can be expressed as the sum of the direct association and the indirect association (*c*_1_ + *a*_1_ × *b*_1_). The 95% confidence intervals for the indirect effects were obtained via bootstrapping with 10,000 iterations; to account for multiple comparisons in this more targeted mediation stage, we applied a Bonferroni correction, reflecting a more conservative adjustment in the context of resampled inference.

After identifying significant mediation associations through morphometric measurements of specific brain regions or neurochemical concentrations, we performed a post-hoc analysis by separating the morphometric measurements into left- and right-hemispheres for associations with OPE metabolite concentrations. We then conducted the same mediation analysis using the separate measurements to improve estimation precision and assess the hemisphere’s associations on the mediation results.

**Fig. 2.**
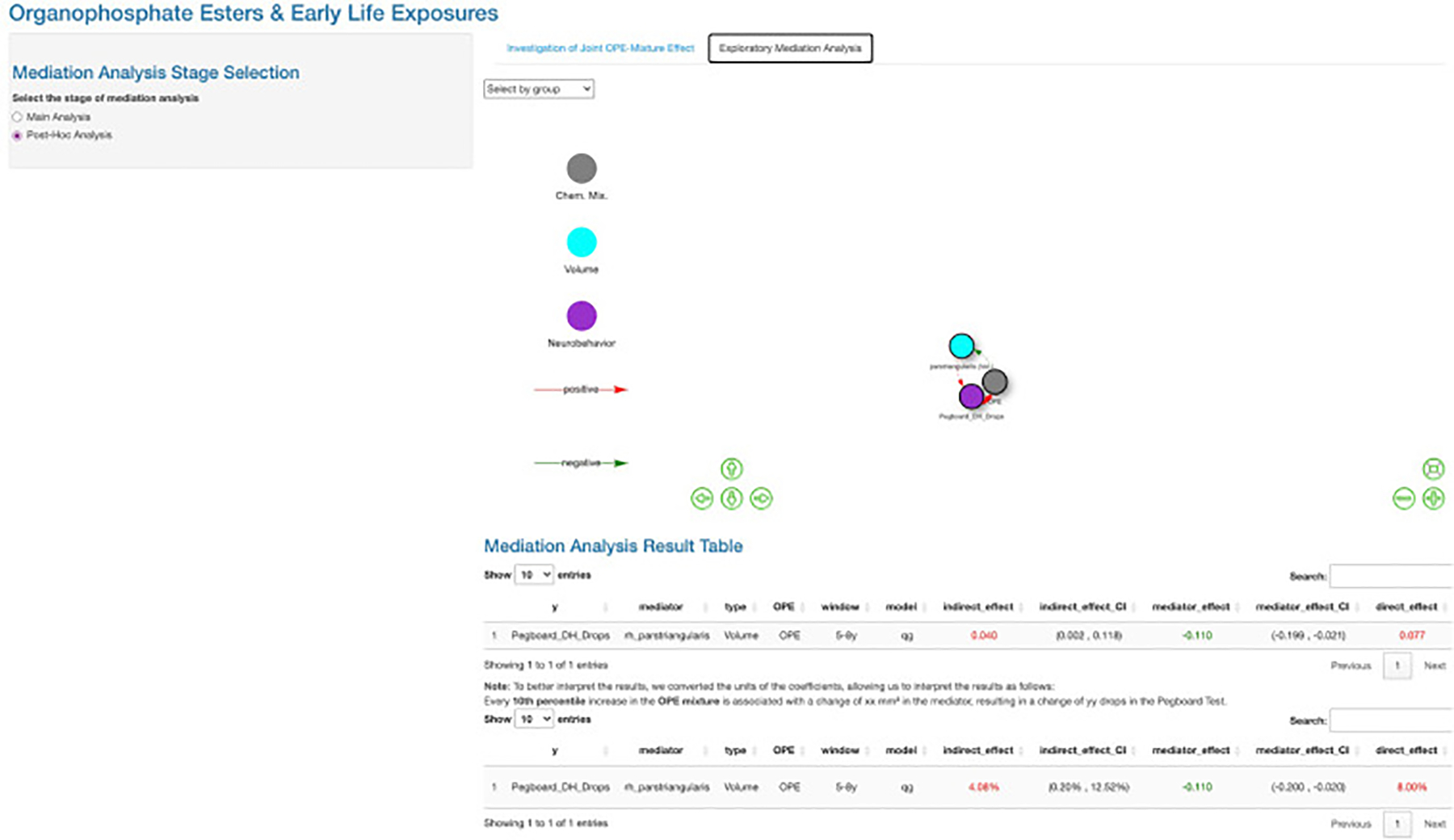
Example page from the R Shiny network application.

Through the two-stage analytical approach, we aimed to establish a comprehensive connection between OPE metabolite concentrations, morphometric measurements, neurochemical concentrations, and neurobehavioral outcomes within each window. Considering the large number of outcomes in this study, we developed an interactive network application using R Shiny that provides end-users with a practical tool to better understand associations between OPE metabolite concentrations and outcomes across windows and between models (Fig. 2). The application is hosted at the following URL: https://zren0723.shinyapps.io/network_app/.

## 3. Results

The characteristics of participants in the neuroimaging and neurobehavioral groups were similar despite sample sizes ranging from 173 to 222 (Table 1). The samples were composed of a slightly higher proportion of females and individuals identifying as White, and most caregivers had completed some college or obtained a bachelor’s degree. The average Home Observation for Measurement of the Environment score was 39. Specific-gravity standardized median urinary concentrations of BCEP, BDCIPP, DNBP, and DPHP were 0.47–1.04 μg/L, 0.59–4.29 μg/L, 0.14–0.33 μg/L, and 1.30–2.63 μg/L, respectively, during the evaluated time windows (Supplementary Table S1). Median scores for neurobehavioral assessments were as follows: ChAMP (97–104), Pegboard (1 drop and 74–81 s), BRIEF (54–56), and BASC (46–52) (Supplementary Table S2).

In the analysis of the OPE-mixture effects, although some associations were observed before multiple-comparison adjustment, none remained statistically significant after correction, likely due to the large number of tests conducted in this exploratory analysis. Table 2 reports the ten smallest unadjusted p-values as exploratory results. Among these ten results, nine were morphometric measurements (CV, CT, or subcortical), and only one was neurobehavior. These results should be considered as exploratory and interpreted with caution. Among these results, 79 had an unadjusted p-value of less than 0.1 and were selected for the exploratory mediation analysis. A full table of the outcomes selected for the exploratory mediation analysis can be found in the supplementary materials (Supplementary Table S3).

In the exploratory mediation analysis, among the 127 mediation models fitted, we identified one borderline significant indirect joint association within the 5–8 year window, mediated through the pars triangularis cortical volume (Table 3). Specifically, within the 5–8 year window, we observed a positive joint association of all four OPE metabolites with the number of drops during the task using the dominant hand in the same window, mediated through the pars triangularis cortical volume. Every decile increase in the mixture of OPE metabolites was associated with a 0.09 (95% CI: −0.15, −0.02) mm^3^ decrease in pars triangularis cortical volume, contributing to a 3.56% (95% CI: 0.00%, 11.29%) increase in the number of drops during the task. This result did not remain statistically significant after adjusting for multiple comparisons. In the post-hoc hemispheric stratification analysis, with associations appearing to be primarily driven by right-hemispheric cortical volumes (Supplementary Table S4). The estimated proportion mediated via the pars triangularis was 27% (indirect/total) for a mixture of four OPEs.

## 4. Discussion

In this study, using quantile g-computation models, we identified evidence that four OPE metabolite concentrations and their mixture, measured during the gestational and childhood periods, were associated with morphometric changes in cortical volume and thickness, involving multiple brain regions at age 12, although these associations did not remain significant after adjusting for multiple comparisons. We further illustrated this with exploratory mediation analyses, showing that later childhood OPE metabolite mixture concentrations at age 5–8 year were associated with adolescent GPT (Pegboard drops) using the dominant hand, although this association did not remain statistically significant after adjusting for multiple comparisons. In the post-hoc analysis, we noted that the association was mainly mediated through the cortical volume of the right hemisphere’s pars triangularis.

In the quantile g-computation models for the OPE metabolite concentration mixture, among the results with the ten smallest unadjusted p-values, the majority of associations were with morphometric features compared with neurobehavioral outcomes, before adjusting for multiple comparisons. These outcomes were associated with exposures across all windows investigated. This suggests that the OPE mixture may affect adolescent neurobehavior and morphometric MRI features, even though: 1) not all associations with adolescent neurobehavior are mediated by morphometric MRI features, potentially because of other neurotoxic mechanisms; 2) some MRI features affected do not translate to measured outcomes we assessed with our neurobehavioral battery. While we assessed neuroimaging, executive function, and behavior at age 12 years, we did not have neuroimaging collected at earlier years of life. Nevertheless, examining mixtures of OPE metabolites offers additional insights into our understanding of joint associations of multiple developmental neurotoxicants. These findings did not remain statistically significant after adjusting for multiple comparisons, as such should be considered as exploratory and need to be externally validated to investigate their potential implications for brain development and neurobehavioral function.

The mediation analyses found that higher OPE metabolite mixture concentrations in the 5–8 year window were associated with an increase in the number of drops during the GPT (Pegboard drops) using the dominant hand, with a mediation decrease in pars triangularis cortical volumes before adjusting for multiple comparisons, with post-hoc analyses indicating the association from the right hemisphere. These results should also be considered exploratory requiring validation in an external dataset.

Although exploratory, these findings suggest that exposure to OPEs potentially affect motor and vision-related neurobehaviors (Bergstrom et al., 2021; Bracci et al., 2016; Kellenbach et al., 2003; Lewis, 2006; Taniguchi et al., 2021). While the pars triangularis is traditionally recognized as involved in speech production, syntax and semantic linguistic processes as part of Broca’s area (Elmer, 2016), it may contribute to cognitive functions including attention and inhibition. Boen et al. (2022) describe how the right inferior frontal gyrus has been associated with the key node for the inhibition of inappropriate motor responses as a component of behavioral control and flexibility. The right pars triangularis, one of three subregions based on cytoarchitecture, was the only subregion identified with structural connectivity to the supplementary motor association area, insula, putamen, caudate, and subthalamic nuclei. Boen et al. speculates that the combination of the pars opercularis and the pars triangularis may be a more appropriate connectivity hub for inhibitory control than the pars opercularis alone. Functional neuroimaging studies with positron emission tomography and functional MRI suggest a role for processing knowledge associated with tool-use ability in cortical regions (Bergstrom et al., 2021; Bracci et al., 2016; Kellenbach et al., 2003; Lewis, 2006; Taniguchi et al., 2021). A study of trained assembly workers demonstrated higher functional connectivity in two regions compared with untrained workers, including one that involved the left supplementary motor area and the right hemisphere pars triangularis of the inferior frontal gyrus (Taniguchi et al., 2021). Untrained workers had stronger connectivity than the trained workers in a network between the left paracentral lobule and right angular gyrus, resulting in speculation that this may reflect a greater reliance on sensorimotor input required for complex tool-use ability (Taniguchi et al., 2021). We are unable to explain the relationship of functional connectivity with cortical volume associated with OPE metabolites across developmental windows in the current study without functional MRI. However, the common involvement of these identified regions with a motor task is noteworthy.

Brain morphometry became accessible with MRI and the development of robust software to segment the brain and parcellate the cortical surface into standardized regions of interest for quantification. Cortical volume is the product of cortical surface area and thickness (Panizzon et al., 2009). Cortical surface area and thickness are distinct features of cortical structure driven by different cellular mechanisms and genetic etiologies (Vijayakumar et al., 2016). Neurons within the cerebral cortex are organized into ontogenetic columns running perpendicular to the brain surface (Mountcastle, 1997). Using the radial unit hypothesis for cellular origin and migration to their cortical location during development, cortical surface area is related to the number of columns, while cortical thickness depends upon the number of cells within a column (Rakic, 1988, 1995, 2007). Cortical brain imaging phenotypes have been studied with twin datasets and genome-wide association studies to estimate the contribution of genetic, common environment, and individual subject variation to variance in cortical morphometry at the region of interest and vertex level (Smith et al., 2024). Four independent longitudinal datasets from three countries (the US, the Netherlands, and Norway) acquired from ages 7 to 29 found consistent developmental trajectories and patterns of change in cortical surface area, thickness, and volume during adolescence. Cortical thickness and volume vary regionally with nonlinear decreases (Tamnes et al., 2017). Heritable genetic factors are thought to influence cortical thickness and surface area significantly more than other factors (Smith et al., 2024). However, experience-dependent shaping of the cortical column architecture and dendritic spines in non-human animal models, along with axonal remodeling, may increase cortical thickness during development (Chklovskii et al., 2004; Hensch, 2005; Mataga et al., 2004; Shaw et al., 2008; Sur and Rubenstein, 2005). Increases in cortical thickness are also attributed to proliferation of myelin into the peripheral cortical neuropil, increased dendritic spines, dendritic and axonal arborization, and glial cell presence (Sowell et al., 2004; Vidal-Pineiro et al., 2020). Inflammatory pathology and neuronal hypertrophy also influence cortical thickness (Phan et al., 2024). Reductions in cortical volume occur with apoptosis, synaptic pruning, and neurodegenerative mechanisms. Environmental chemicals, such as OPEs, have largely been ignored in neuroimaging investigations evaluating brain morphometry, especially in children and adolescents. However, recent longitudinal cohort studies evaluating brain development in typical populations have begun to evaluate the effects of environmental toxicants. Leveraging the Adolescent Brain Cognitive Development Study data, Marshall et al. (2021) found that children ages 9 and 10 years who lived in neighborhoods with greater risks of environmental lead exposure from older housing showed smaller volumes of the corpus callosum. Using ABCD data collected longitudinally from ages 9–13 years across the US, de Jesus et al. (2025) reported that the higher particulate matter less than 2.5 μm exposure had greater cortical thinning in the occipital and temporal lobes and was further exacerbated by neighborhood disadvantage.

Witchey et al. recently identified multiple modes of action for how gestational exposure to OPEs can interrupt neurodevelopment (Witchey et al., 2023). Novel lipidomic analyses of cortical tissues found upregulation of genes by OPEs that result in the accumulation of brain ceramides which disrupt mitochondrial function. While we are currently unable to confirm if the metabolite profile of the HOME Study cohort is consistent with the models evaluated, other mechanisms for dysregulation of mitochondrial function and disruption of cholinergic and glutamatergic systems were also observed (Newell et al., 2023; Witchey et al., 2023). Specifically, OPEs disrupt mitochondrial ATP synthesis coupled electron transport, respiratory electron transport chain processes and NADH dehydrogenase complex assembly (Witchey et al., 2023). These pathways are associated with nervous system development, synaptic transmission, and axonogenesis, and interfere with glutamatergic and cholinergic synapse activity and function (Witchey et al., 2023). Cortical refinement such as dendrite stabilization, neurogenesis and synaptogenesis relies on acetylcholine (Witchey et al., 2023). Slotkin et al. when evaluating *in vitro* models with embryonic rat neural stem cells, reported OPEs changed the neuronal phenotype from the formation of neurons instead favoring glia to produce an increased glia/neuron ratio (Slotkin et al., 2017). Further, in rat neuronotypic PC12 cells, the OPE exposure enhanced neurodifferentiation into acetylcholine and dopamine phenotypes. These modes of action disrupted by OPEs support morphometric variations in brain structure, specifically changes in cortical volume and thickness (Slotkin et al., 2017).

Our study has several strengths, including: 1) exploring the associations of OPE metabolites on neurochemical concentrations, morphometric measurements, and neurobehavioral outcomes collectively; 2) considering the collective associations of OPE metabolites in different windows through a quantile g-computation model; 3) investigating the associations of OPE metabolites on neurobehavioral outcomes through changes in morphometry or neurochemical concentrations by conducting mediation analysis; and 4) creating an interactive chemical mixture associations network through a Shiny app.

However, our study does have some limitations. In both stages of the analysis, we conducted multiple hypothesis tests across a large array of outcomes and our modest sample size may have severely limited statistical power. The exploratory associations identified in this study’s morphometric measurements require further testing in additional studies with a larger sample size. The morphometric associations may not capture the individual and collective associations of OPE metabolites in development if exposure does not induce structural changes. Instead, a more dynamic measure, such as resting-state functional MRI, may be more insightful for revealing how brain networks have been adapted in response to OPE exposures, rather than anatomically changed with a discrete volume or thickness measure. The neurochemical acquisition was limited by the sampling of only one anatomical location, so while it is a temporally more dynamic measure than morphometric measures such as volume or thickness, its location was not specifically optimized for OPE exposure associations. We previously identified that childhood OPE metabolite concentrations were related to cognitive deficits at age 8 years among children from a lower socioeconomic background within the HOME Study (Percy et al., 2022). Future studies will benefit from further examining whether effect modification by social determinants of health on neuroimaging outcomes exists and if these account for health disparities in neurobehavioral outcomes.

## 5. Conclusions

In summary, we explored the relationships between early life OPE metabolite urinary concentrations, morphometric MRI features, intrinsic neurochemistry as measured by MRS, motor function, executive function, and problem behaviors in adolescents. We identified an association between the OPE metabolite mixture at the 5–8 year window and a reduction in the volume of the pars triangularis cortical region, particularly in the right hemisphere, which may mediate the association between OPE metabolites and visual motor function assessed with the grooved pegboard test in exploratory mediation analyses, but did not remain statistically significant after adjusting for multiple comparisons. Additional associations between OPE metabolite concentrations and outcomes were identified in the first analysis, but these did not remain statistically significant after adjustment for multiple testing. This may reflect limited statistical power under conservative correction and modest effects in this sample. Future research with larger and more diverse populations that incorporate mixture-based analyses, consider developmental windows of susceptibility, and include earlier multi-modal neuroimaging, such as functional MRI, to better understand the potential developmental neurotoxicity of OPEs.

## Supplementary Material

Supplement 1

## Figures and Tables

**Table 1.** Demographic table of participants included in the study related to neurobehavioral outcomes.

Single Subgroup	MRS (N=173)	Morphometry (N=177)	ChAMP (N=217)	GPT (N=213)	BRIEF (N=219)	BASC (N=222)
**Adolescent-Sex**						
Male	76 (44)	77 (44)	97 (45)	98 (46)	98 (45)	96 (43)
Female	97 (56)	100 (56)	120 (55)	115 (54)	121 (55)	126 (57)
**Maternal Race**						
Non-white	76 (44)	78 (44)	92 (42)	90 (42)	87 (40)	91 (41)
White	97 (56)	99 (56)	125 (58)	123 (58)	132 (60)	131 (59)
**HOME Score**	38.68 (5.45)	38.65 (5.43)	39.12 (5.16)	39.06 (5.21)	39.28 (5.05)	39.24 (5.13)
**Maternal Education**						
High School or Less	46 (27)	46 (26)	52 (24)	51 (24)	53 (24)	52 (23)
Some college	53 (31)	55 (31)	64 (29)	62 (29)	58 (26)	62 (28)
Bachelor	48 (28)	49 (28)	65 (30)	64 (30)	67 (31)	68 (31)
Graduate	26 (15)	27 (15)	36 (17)	36 (17)	41 (19)	40 (18)

Combined Subgroup	Mediation: ChAMP (N=168)	Mediation: GPT (N=165)	Mediation: BRIEF (N=165)	Mediation: BASC (N=167)
**Adolescent-Sex**				
Male	74 (44)	74 (45)	73 (44)	71 (43)
Female	94 (56)	91 (55)	92 (56)	96 (57)
**Maternal Race**				
Non-white	74 (44)	72 (44)	70 (42)	72 (43)
White	94 (56)	93 (56)	95 (58)	95 (57)
**HOME Score**	38.68 (5.46)	38.67 (5.48)	38.73 (5.38)	38.78 (5.43)
**Maternal Education**				
High School or Less	44 (26)	43 (26)	45 (27)	43 (26)
Some college	52 (31)	50 (30)	47 (28)	50 (30)
Bachelor	47 (28)	47 (28)	47 (28)	48 (29)
Graduate	25 (15)	25 (15)	26 (16)	26 (16)

Note: MRS: magnetic resonance spectroscopy; ChAMP: The Child and Adolescent Memory Profile; GPT: grooved pegboard test; BRIEF: Behavior Rating Inventory of Executive Functioning, second edition; BASC: Behavioral Assessment System for Children, third edition; N: number of participants; Values for Home Observation for Measurement of the Environment (HOME) Scores are given as mean (standard deviation); Values for other variables are given as count (proportion).

**Table 2.** OPE Mixture Analysis Results

Outcome	Type	Window	Estimate (90% CI)	p-value	Adjusted p-value
**Superior frontal**	CV	Birth	0.33 (0.14, 0.53)	0.005	0.58
**Pars triangularis**	CV	5–8y	−0.10 (−0.16, −0.04)	0.005	0.58
**Surface area**	Sub	Birth	816.99 (334.50, 1299.48)	0.006	0.58
**Precentral**	CV	3y	0.19 (0.08, 0.30)	0.007	0.58
**GPT Drops NDH**	Neuro	5–8y	0.15 (0.06, 0.24)	0.008	0.58
**Posterior cingulate**	CV	Birth	0.06 (0.02, 0.09)	0.009	0.58
**LH frontal pole**	CT	Birth	−0.04 (−0.07, −0.02)	0.009	0.58
**Isthmus cingulate**	CV	3y	0.04 (0.02, 0.07)	0.013	0.58
**Paracentral**	CV	3y	0.06 (0.02, 0.11)	0.013	0.58
**LH posterior cingulate**	CT	Gestational	0.02 (0.01, 0.03)	0.014	0.58

Window refers to the time period of the exposure; Estimate (CI) OPE’s unadjusted association with the outcome and 90% confidence interval; p-value unadjusted p-value for association; Adjusted p-value: BH adjusted p-value; Outcome represents the outcome of the model; Type refers to the type of the outcome; CV: Cortical volume in mm^2^ units; Sub: Subcortical volume in mm^2^ units; CT: Cortical thickness in mm units; Neuro: Neurobehavior; GPT Drops NDH: number of drops during task using the non-dominant hand obtained from the grooved pegboard test; RH: right hemisphere; LH: left hemisphere

**Table 3.** Mediation analysis results within the 5–8 year window.

Component	Estimate (95% CI)	Estimate (Bonferroni-adjusted 95% CI)
**Outcome**	GPT Drops	GPT Drops
**Exposure**	OPE mixture	OPE mixture
**Mediator**	Pars triangularis	Pars triangularis
**Effect of exposure on mediator**	−0.09 (−0.15, −0.02)	−0.09 (−0.20, 0.03)
**Indirect effect (%)**	3.56 (0.00, 11.29)	3.56 (−3.44, 23.12)
**Direct effect (%)**	8.87 (−3.73, 23)	8.87 (−12.80, 35.93)
**Total effect (%)**	12.75 (−0.2, 30.21)	12.75 (−11.04, 51.44)

Outcome represents the neurobehavioral outcome; Exposure represents the OPE mixture; Mediator represents the morphometric outcome identified; Indirect effect (%) represents the estimated indirect association ($a_{1}\times b_{1}$); Direct effect (%) represents the estimated direct association ($c_{1}$). Total effect (%) represents the estimated total association ($c_{1}+a_{1}\times b_{1}$); GPT Drops represents the number of drops during task using the dominant hand obtained from the grooved pegboard test. The 95% CI represents the unadjusted 95% confidence interval; Bonferroni-adjusted 95% CI accounts for multiple testing.

## Data Availability

The data that has been used is confidential.

## Appendix A. Supplementary data

Supplementary data to this article can be found online at https://doi.org/10.1016/j.envres.2026.124509.
