# Supplementary material for: Identifying associations and key early life exposure windows for organophosphate esters and adolescent brain morphometry in the HOME study
Source: Environ Res. Author manuscript; Available in PMC 2026 Jul 27. (PMC13402942; doi:10.1016/j.envres.2026.124509)
Supplement: Supplement 1 [file NIHMS2197404-supplement-Supplement_1.docx]

**Supplementary Materials**

**Identifying Associations and Key Early Life Exposure Windows for Organophosphate Esters and Adolescent Brain Morphometry in the HOME Study**

Zheng Ren^1,2^, Aimin Chen^3^, Jagadeesh Puvvula^3^, Yingying Xu^4^, Ethan Bouche^2,3^, Antonia M. Calafat^5^, Alex D. Edmondson^6,7^, Kristin A. Linn^2,3^, Russell T. Shinohara^2,3,8^, Maria Ospina^5^, Zana Percy^7^, Ann M. Vuong^9^, Joseph M. Braun^10^, Bruce P. Lanphear^11,12^, Kimberly Yolton^4^, Quy Cao^2,3^*, Kim M. Cecil^4,6,7^*

1. Department of Biostatistics, Johns Hopkins Bloomberg School of Public Health
2. Penn Statistics in Imaging and Visualization Center, Department of Biostatistics, Epidemiology, and Informatics, University of Pennsylvania, Philadelphia, PA, USA
3. Department of Biostatistics, Epidemiology and Informatics, Perelman School of Medicine, University of Pennsylvania, Philadelphia, PA, USA
4. Department of Pediatrics, Cincinnati Children's Hospital Medical Center, University of Cincinnati College of Medicine, Cincinnati, OH, USA
5. National Center for Environmental Health, U.S. Centers for Disease Control and Prevention, Atlanta, GA
6. Department of Radiology, Cincinnati Children's Hospital Medical Center, University of Cincinnati College of Medicine, Cincinnati, OH, USA
7. Department of Environmental & Public Health Sciences, College of Medicine, University of Cincinnati, Cincinnati, OH
8. Center for Biomedical Image Computing and Analytics, University of Pennsylvania, Philadelphia, PA, USA
9. Department of Epidemiology and Biostatistics, School of Public Health, University of Nevada Las Vegas, Las Vegas, NV, USA
10. Department of Epidemiology, Brown University, Providence, RI, USA
11. Faculty of Health Sciences, Simon Fraser University, Burnaby, BC, Canada
12. Child and Family Research Institute, BC Children's Hospital, Vancouver, BC, Canada

**Supplementary Table S1.** Descriptive statistics of OPE metabolite specific-gravity standardized concentrations (µg/L) at different time points for the final study sample.

| OPE | N | % < LOD | Quantile | | | IQR |
| --- | --- | --- | --- | --- | --- | --- |
|  |  |  | **25** | **50** | **75** |  |
| BCEP |  |  |  |  |  |  |
| 16w | 236 | 11 | 0.33 | 0.62 | 1.14 | 0.82 |
| 26w | 218 | 16 | 0.23 | 0.47 | 1.06 | 0.83 |
| Birth | 204 | 11 | 0.27 | 0.52 | 0.95 | 0.68 |
| 1y | 147 | 5 | 0.41 | 0.83 | 2.22 | 1.81 |
| 2y | 139 | 9 | 0.50 | 0.97 | 2.62 | 2.13 |
| 3y | 160 | 4 | 0.45 | 1.04 | 2.68 | 2.23 |
| 5y | 157 | 6 | 0.38 | 0.77 | 1.76 | 1.38 |
| 8y | 189 | 21 | 0.34 | 0.72 | 1.46 | 1.12 |
| BDCIPP |  |  |  |  |  |  |
| 16w | 234 | 3 | 0.44 | 0.77 | 1.49 | 1.05 |
| 26w | 218 | 10 | 0.28 | 0.59 | 0.97 | 0.68 |
| Birth | 200 | 7 | 0.32 | 0.62 | 1.28 | 0.96 |
| 1y | 149 | 1 | 0.68 | 1.29 | 3.09 | 2.41 |
| 2y | 143 | 1 | 1.05 | 1.95 | 4.35 | 3.30 |
| 3y | 160 | 1 | 1.51 | 3.33 | 6.92 | 5.41 |
| 5y | 155 | 1 | 1.47 | 3.11 | 8.08 | 6.61 |
| 8y | 192 | 0 | 2.04 | 4.29 | 7.96 | 5.92 |
| DNBP |  |  |  |  |  |  |
| 16w | 235 | 14 | 0.16 | 0.24 | 0.35 | 0.20 |
| 26w | 214 | 23 | 0.12 | 0.21 | 0.31 | 0.18 |
| Birth | 194 | 43 | 0.09 | 0.14 | 0.20 | 0.11 |
| 3y | 126 | 13 | 0.18 | 0.33 | 0.68 | 0.50 |
| 5y | 155 | 15 | 0.17 | 0.27 | 0.44 | 0.28 |
| 8y | 192 | 35 | 0.10 | 0.17 | 0.27 | 0.17 |
| DPHP |  |  |  |  |  |  |
| 16w | 237 | 1 | 1.01 | 1.62 | 2.89 | 1.88 |
| 26w | 218 | 2 | 0.78 | 1.30 | 2.17 | 1.39 |
| Birth | 204 | 0 | 0.91 | 1.60 | 3.12 | 2.22 |
| 1y | 148 | 0 | 1.28 | 2.15 | 3.64 | 2.37 |
| 2y | 142 | 0 | 1.58 | 2.59 | 4.21 | 2.63 |
| 3y | 160 | 0 | 1.67 | 2.61 | 4.49 | 2.82 |
| 5y | 158 | 0 | 1.53 | 2.63 | 6.40 | 4.87 |
| 8y | 192 | 0 | 1.48 | 2.43 | 4.17 | 2.69 |

Note: OPE: organophosphate ester; LOD: limit of detection; µg/L: micrograms per liter; w: weeks; y: years; DPHP: diphenyl phosphate; DNBP: di-n-butyl phosphate; BDCIPP: bis(1,3-dichloro-2-propyl) phosphate; BCEP: bis-2-chloroethyl phosphate; LOD was 0.1 µg/L for all four OPE metabolites evaluated.

**Supplementary Table S2.** Descriptive statistics for four types of neurobehavioral test for the final study sample. Abbreviations: ChAMP: Child and Adolescent Memory Profile; BRIEF: Behavior Rating Inventory of Executive Function, Second Edition; Behavior Assessment System for Children, Third Edition; DH: dominant hand; NDH: non-dominant hand; BRI: Behavior Regulation Index; CRI: Cognitive Regulation Index; ERI: Emotion Regulation Index; GEC: Global Executive Composite; ESI:Emotional Symptoms Index; FII: Functional Impairment Index; IHI: Inattention Hyperactivity Index; INZ: Internalizing Problems Index; PAI: Personal Adjustment Index; SPI: School Problems Index

| Neurobehavior Test | Quantile | | | IQR | SD |
| --- | --- | --- | --- | --- | --- |
|  | **25** | **50** | **75** |  |  |
| ChAMP | 92 | 98 | 106 | 14 | 13.02 |
| Index Delayed | 92 | 98 | 106 | 14 | 12.26 |
| Index Immediate | 89 | 97 | 106 | 17 | 12.72 |
| Index Total Memory | 90 | 98 | 106 | 16 | 13.76 |
| Index Verbal | 95 | 104 | 113 | 18 | 12.29 |
| Index Visual | 86 | 93 | 100 | 14 | 13.02 |
| Pegboard |  |  |  |  |  |
| DH Drops | 0 | 1 | 1 | 1 | 0.97 |
| DH Seconds | 67 | 74 | 85 | 18 | 14.87 |
| NDH Drops | 0 | 1 | 2 | 2 | 1.13 |
| NDH Seconds | 73 | 81 | 90 | 17 | 18.19 |
| BRIEF |  |  |  |  |  |
| Adolescent BRI | 47 | 55 | 62.25 | 15.25 | 10.02 |
| Adolescent CRI | 48 | 56 | 62 | 14 | 9.40 |
| Adolescent ERI | 47 | 54 | 62 | 15 | 9.65 |
| Adolescent GEC | 49 | 55 | 62 | 13 | 9.60 |
| BASC |  |  |  |  |  |
| Adolescent ESI | 42 | 46 | 54 | 12 | 10.43 |
| Adolescent FII | 42 | 48 | 55.5 | 13.5 | 11.14 |
| Adolescent IHI | 42 | 49 | 58 | 16 | 10.93 |
| Adolescent INZ | 42 | 47 | 55 | 13 | 10.84 |
| Adolescent PAI | 46 | 52 | 57 | 11 | 9.12 |
| Adolescent SPI | 44 | 49 | 57 | 13 | 10.28 |

**Supplementary Table S3.** Outcomes selected for use in exploratory mediation analysis. Window refers to the time period of the exposure; Estimate (CI) OPE’s unadjusted association with the outcome and 90% confidence interval; p-value unadjusted p-value for association; Adjusted p-value: BH adjusted p-value; Outcome represents the outcome of the model; Type refers to the type of the outcome; CV: Cortical volume; Sub: Subcortical volume; Neuro: Neurobehavior; Pegboard_NDH_Drops: number of drops during task using the non-dominant hand obtained from the grooved pegboard test

| Outcome | Type | Window | Estimate(90% CI) | P-value | Adjusted  P-value |
| --- | --- | --- | --- | --- | --- |
| Cho | metabolites | prenatal | -0.02(-0.03, 0) | 0.038 | 0.58 |
| paracentral | CV | prenatal | 0.05(0.01, 0.08) | 0.038 | 0.58 |
| precentral | CV | prenatal | 0.13(0.03, 0.23) | 0.040 | 0.58 |
| superiortemporal | CV | prenatal | 0.11(0.01, 0.2) | 0.060 | 0.62 |
| rh_caudalanteriorcingulate | CV | birth | 0.05(0.01, 0.09) | 0.050 | 0.58 |
| rh_frontalpole | CV | birth | 0.02(0.01, 0.04) | 0.031 | 0.58 |
| lh_temporalpole | CV | birth | -0.03(-0.06, 0) | 0.097 | 0.65 |
| caudalmiddlefrontal | CV | birth | 0.11(0.03, 0.19) | 0.028 | 0.58 |
| inferiorparietal | CV | birth | 0.15(0.01, 0.29) | 0.072 | 0.62 |
| lateralorbitofrontal | CV | birth | 0.09(0.02, 0.16) | 0.032 | 0.58 |
| middletemporal | CV | birth | 0.11(0.01, 0.22) | 0.076 | 0.62 |
| paracentral | CV | birth | 0.05(0.01, 0.09) | 0.026 | 0.58 |
| postcentral | CV | birth | 0.1(0.01, 0.18) | 0.066 | 0.62 |
| posteriorcingulate | CV | birth | 0.06(0.02, 0.09) | 0.009 | 0.58 |
| precentral | CV | birth | 0.15(0.04, 0.26) | 0.031 | 0.58 |
| rostralanteriorcingulate | CV | birth | 0.04(0, 0.08) | 0.066 | 0.62 |
| superiorfrontal | CV | birth | 0.33(0.14, 0.53) | 0.005 | 0.58 |
| superiortemporal | CV | birth | 0.1(0, 0.2) | 0.090 | 0.65 |
| transversetemporal | CV | birth | 0.01(0, 0.03) | 0.092 | 0.65 |
| rh_temporalpole | CV | 3y | 0.05(0.01, 0.09) | 0.030 | 0.58 |
| cuneus | CV | 3y | 0.05(0.01, 0.1) | 0.069 | 0.62 |
| inferiortemporal | CV | 3y | 0.15(0.02, 0.28) | 0.063 | 0.62 |
| isthmuscingulate | CV | 3y | 0.04(0.02, 0.07) | 0.013 | 0.58 |
| lateraloccipital | CV | 3y | 0.14(0.01, 0.27) | 0.073 | 0.62 |
| lingual | CV | 3y | 0.11(0.02, 0.2) | 0.051 | 0.58 |
| medialorbitofrontal | CV | 3y | 0.06(0.01, 0.12) | 0.045 | 0.58 |
| paracentral | CV | 3y | 0.06(0.02, 0.1) | 0.013 | 0.58 |
| pericalcarine | CV | 3y | 0.05(0.01, 0.09) | 0.038 | 0.58 |
| posteriorcingulate | CV | 3y | 0.04(0, 0.08) | 0.081 | 0.63 |
| precentral | CV | 3y | 0.19(0.08, 0.3) | 0.007 | 0.58 |
| precuneus | CV | 3y | 0.15(0.05, 0.25) | 0.016 | 0.58 |
| superiorfrontal | CV | 3y | 0.29(0.06, 0.52) | 0.039 | 0.58 |
| parstriangularis | CV | 5-8y | -0.1(-0.16, -0.04) | 0.005 | 0.58 |
| pericalcarine | CV | 5-8y | 0.05(0.01, 0.09) | 0.051 | 0.58 |
| lh_posteriorcingulate | CT | prenatal | 0.02(0.01, 0.03) | 0.014 | 0.58 |
| lh_frontalpole | CT | birth | -0.04(-0.06, -0.01) | 0.009 | 0.58 |
| lh_parsorbitalis | CT | birth | -0.03(-0.04, -0.01) | 0.015 | 0.58 |
| rh_parsorbitalis | CT | birth | -0.02(-0.04, 0) | 0.096 | 0.65 |
| lh_rostralanteriorcingulate | CT | birth | -0.02(-0.03, 0) | 0.093 | 0.65 |
| entorhinal | CT | birth | -0.03(-0.05, 0) | 0.076 | 0.62 |
| superiorparietal | CT | birth | -0.01(-0.02, 0) | 0.098 | 0.65 |
| insula | CT | birth | -0.01(-0.02, 0) | 0.098 | 0.65 |
| rh_isthmuscingulate | CT | 3y | 0.02(0, 0.03) | 0.095 | 0.65 |
| rh_posteriorcingulate | CT | 3y | 0.02(0, 0.03) | 0.020 | 0.58 |
| rh_temporalpole | CT | 3y | 0.03(0, 0.06) | 0.075 | 0.62 |
| cuneus | CT | 3y | 0.01(0, 0.02) | 0.047 | 0.58 |
| inferiorparietal | CT | 3y | 0.01(0, 0.02) | 0.038 | 0.58 |
| lingual | CT | 3y | 0.01(0, 0.02) | 0.071 | 0.62 |
| paracentral | CT | 3y | 0.02(0.01, 0.03) | 0.016 | 0.58 |
| precentral | CT | 3y | 0.01(0, 0.03) | 0.030 | 0.58 |
| rh_frontalpole | CT | 5-8y | 0.03(0, 0.05) | 0.058 | 0.62 |
| lh_posteriorcingulate | CT | 5-8y | -0.02(-0.03, 0) | 0.031 | 0.58 |
| CC_Posterior | Sub | prenatal | -11.83(-20.55, -3.11) | 0.027 | 0.58 |
| CC_Anterior | Sub | prenatal | -9.88(-19.02, -0.74) | 0.077 | 0.62 |
| BrainSegVol | Sub | birth | 7248(1215.27, 13280.73) | 0.050 | 0.58 |
| TotalGrayVol | Sub | birth | 3891.01(764.89, 7017.14) | 0.043 | 0.58 |
| SupraTentorialVol | Sub | birth | 7118.27(1371.9, 12864.63) | 0.044 | 0.58 |
| Accumbens_area | Sub | birth | 7.85(0.87, 14.83) | 0.067 | 0.62 |
| CortexVol | Sub | birth | 1895.14(458, 3332.28) | 0.032 | 0.58 |
| surface_area | Sub | birth | 816.99(334.5, 1299.48) | 0.006 | 0.58 |
| total_brain | Sub | birth | 7181.68(1224.27, 13139.1) | 0.049 | 0.58 |
| CC_Central | Sub | 3y | 19.22(6.61, 31.84) | 0.014 | 0.58 |
| CC_Mid_Anterior | Sub | 3y | 18.32(3.39, 33.25) | 0.046 | 0.58 |
| TotalGrayVol | Sub | 3y | 4645.27(1001.85, 8288.68) | 0.038 | 0.58 |
| SupraTentorialVol | Sub | 3y | 6663.6(249.05, 13078.15) | 0.091 | 0.65 |
| Thalamus_Proper | Sub | 3y | 53.17(8.75, 97.59) | 0.052 | 0.58 |
| Amygdala | Sub | 3y | 16.54(1.37, 31.72) | 0.076 | 0.62 |
| CortexVol | Sub | 3y | 2248.54(569.26, 3927.81) | 0.030 | 0.58 |
| CC_Anterior | Sub | 5-8y | 12.23(0.78, 23.68) | 0.082 | 0.63 |
| choroid_plexus | Sub | 5-8y | 15.6(4.71, 26.48) | 0.020 | 0.58 |
| BRIEF_Child_ERI | Neuro | prenatal | 0.62(0.05, 1.19) | 0.077 | 0.62 |
| BASC_Child_SPI_GC_T | Neuro | prenatal | -0.83(-1.43, -0.24) | 0.021 | 0.58 |
| ChAMP_Index_Verbal | Neuro | birth | 1(0.05, 1.96) | 0.085 | 0.65 |
| Pegboard_DH_Seconds | Neuro | birth | -1.24(-2.16, -0.32) | 0.027 | 0.58 |
| Pegboard_NDH_Seconds | Neuro | birth | -1.28(-2.48, -0.08) | 0.082 | 0.63 |
| Pegboard_NDH_Drops | Neuro | birth | -0.08(-0.16, -0.01) | 0.075 | 0.62 |
| Pegboard_NDH_Seconds | Neuro | 3y | -1.39(-2.52, -0.26) | 0.045 | 0.58 |
| Pegboard_DH_Drops | Neuro | 5-8y | 0.08(0.01, 0.15) | 0.058 | 0.62 |
| Pegboard_NDH_Drops | Neuro | 5-8y | 0.15(0.06, 0.24) | 0.008 | 0.58 |

**Supplementary Table S4.** Post-Hoc Mediation analysis results within the 5-8 year window.

Outcome represents the neurobehavioral outcome; Exposure represents the OPE mixture; Mediator represents the morphometric outcome identified; Indirect effect (%) represents the estimated indirect association ($a_{1}\times b_{1}$); Direct effect (%) represents the estimated direct association ($c_{1}$). Total effect (%) represents the estimated total association ($c_{1}+ a_{1}\times b_{1}$); GPT Drops represents the number of drops during the task using the dominant hand obtained from the grooved pegboard test. The 95% CI represents the unadjusted 95% confidence interval; RH-: right hemisphere.

| Component | Estimate (95% CI) |
| --- | --- |
| Outcome | GPT Drops |
| Exposure | OPE mixture |
| Mediator | RH-Pars triangularis |
| Effect of exposure on mediator | -0.11 (-0.20, -0.02) |
| Indirect effect (%) | 4.08 (0.20, 12.52) |
| Direct effect (%) | 8.00 (-4.69, 22.38) |
| Total effect (%) | 13.54 (-5.92, 37.71) |
